# Association between Hypertension, Antihypertensive Drugs, and Osteoporosis in Postmenopausal Syrian Women: A Cross-Sectional Study

**DOI:** 10.1155/2020/7014212

**Published:** 2020-02-19

**Authors:** Nermeen Hijazi, Zaynab Alourfi

**Affiliations:** ^1^Internal Medicine Department—Endocrinology, Damascus University, Damascus, Syria; ^2^Faculty of Medicine, Syrian Private University (SPU), Damascus, Syria

## Abstract

**Background:**

Osteoporosis and hypertension are frequent and often coexisting diseases among the elderly. Recent studies suggested that both diseases may share the same etiopathology. Moreover, the treatment of hypertension can affect the bone mineral density and worsen osteoporosis. The aim of this cross-sectional study was to assess the prevalence of low bone mass and osteoporosis in postmenopausal Syrian women and investigate their relationship with hypertension and antihypertensive drugs.

**Methods:**

813 postmenopausal women were involved in this cross-sectional study, aged between 40 and 96 yrs. Their menopause duration ranged between 1 and 43 yrs. Bone mineral density was measured using a dual-energy X-ray absorptiometry at the total lumbar spine (L1-L4) and left hip. T-score values were used to determine the diagnosis of osteoporosis. The existence of HTN was defined as blood pressure ≥130/85 mmHg or a history of hypertension medication.

**Results:**

Using the world health organization criteria, 24% had osteoporosis and 45.2% had low bone mass. The incidence of osteoporosis and low bone mass significantly increased with age and menopause duration and decreased with BMI. Prevalence of hypertension was almost equal among the women who had or did not have osteoporosis. However, hypertensive women who used thiazides or beta blockers had higher values of total lumbar BMD compared with the women who did not.

**Conclusion:**

Hypertension in postmenopausal Syrian women aged over 40 was not found to be associated with osteoporosis. However, the mean total lumbar BMD of the hypertensive women who took thiazide diuretics or beta blocker was found to be increased significantly comparing to the women who did not take either.

## 1. Introduction

The diagnosis of both osteoporosis and HTN has been increasing globally due to the increased number of people aged over 50 yrs driven by the increasing longevity [1–3].

It has been estimated that 50% of women over 50 yrs had low bone mass according to the national health nutrition examination survey, and about 25% of women over 60 yrs had osteoporosis [4], and 20% to 40% is the worldwide prevalence of hypertension [5, 6].

The increased comorbidities and mortality associated with these two diseases illustrate their clinical risk.

On one hand, HTN is a major risk factor for ischemic heart disease, renal failure, and other ischemic vascular diseases [7–9], and as a result, it accounts for 1–4% of all causes of death [6–10].

On the other hand, osteoporotic fractures are important causes of disability [3]. Hip fracture is associated with a 20% excess mortality one year following the fracture [11].

Recently, many epidemiological and biological studies suggested that both HTN and osteoporosis share the same etiopathology, involving low calcium intake and level, vitamin D and vitamin K deficiency, and low or very high levels of nitric oxide [12].

The prevalence of low bone mass, osteoporosis, and hypertension among postmenopausal Syrian women was determined in this study, and the association between osteoporosis, hypertension, and antihypertensive drugs was explored.

## 2. Methods

### 2.1. Study Population

A cross-sectional study was performed between November 2018 and March 2019 at Al-Mouwasat University Hospital, Damascus, Syria. Participants were postmenopausal women aged ≥40 yrs. Subjects who were previously diagnosed with osteoporosis, chronic kidney disease (glomerular filtration rate (GFR) <30 mL/min/1.73 m^2^), chronic liver disease, advanced heart disease, metabolic or inherited bone disease, such as hyperparathyroidism or hypoparathyroidism, Paget disease, osteomalacia, or osteogenesis imperfecta, Cushing syndrome, hyperthyroidism, or took medication that proved to increase bone mineral density (such as bisphosphonate, hormone replacement therapy, selective estrogen receptor modulator, strontium ranelate, calcitonin, and PTH Analog) were excluded.

HTN was defined as blood pressure ≥130/85 mmHg or a history of hypertension medication.

Information was collected by a questionnaire included age, menopause duration, work, exercise, cigarette smoking, and regular alcohol consumption.

Weight (with light clothes) and barefoot height were measured using the Seca Scale Model 713 device (Boian Surgical, Padstow, Australia). Body mass index was calculated by the equation (BMI = weight (kg)/height (m^2^)). Participants were categorized according to the criteria by the World Health Organization (WHO) as follows: BMI <25 kg/m^2^ for normal weight, 25 ≤ BMI < 30 kg/m^2^ for overweight, and BMI ≥30 kg/m^2^ for obesity.

Total body measurement of BMD was made using a dual-energy X-ray absorptiometry (Medilink, MEDIX DR VER v4.0.3) at the total lumbar spine (L1-L4) and left hip. If a fracture or degeneration was recorded at one or two lumbar vertebrae, those vertebrae were excluded from the DXA report, and the diagnosis was made according to the rest of the lumbar vertebrae. If three or more vertebrae were affected, the lumbar BMD was excluded from the report, and the participant was excluded from the study [13]. The left hip was not scanned when a positive history of fracture or surgery was present, and the right hip was scanned instead. The scanner was used by the same well-trained nurse and calibrated daily against the standard calibration block supplied by the manufacturer. Osteoporosis was diagnosed according to the WHO criteria, using T-score values shown in Table 1.

Data were analyzed using the SPSS software version 23.0 (IBM, Armonk, New York, USA), *p* values <0.05 were considered statistically significant. Mean and standard deviation were calculated. The chi-square test was used for nominal data and independent-samples T-test for continuous normally disturbed variables.

## 3. Results

813 postmenopausal women were included, their mean age was 58.92 ± 8.6 yr, and their mean menopause duration was 10.1 ± 8.3 yr, with an average height of 152.16 cm ± 6.38 cm, weight 77.09 kg ± 14.51 kg, BMI 33.35 kg/m^2^ ± 6.13 kg/m^2^, total lumbar BMD 0.905 g/cm^2^ ± 0.162 g/cm^2^, and femoral neck BMD 0.928 g/cm^2^ ± 0.148 g/cm^2^ (Table 2). 182(22.3%) women were smokers, 288 (35.42%) women regularly exercised, and 232 (28.5%) were workers. None of the participants consumed alcohol regularly as it is an unpopular habit in the Syrian community. Osteoporosis was found in 195 women (24%), and low bone mass was found in 368 women (45.2%) while normal bone density was found in 250 women (30.8%).  Table 3 shows the general characteristics of the participants of the study.

The incidence of osteoporosis and low bone mass increased significantly with age and menopause duration (*p* : 0.0001) (Table 4).

Obesity, overweight, and normal weight prevalence were 69.6%, 23.1%, and 7.3%, respectively. The normal weight group had the highest osteoporosis incidence with a significant difference (*p* : 0.0001) Table 4.

Among the 813 women included, 387 (47.7%) had HTN. (93.1%) Of them were treated with one or more antihypertensive drugs and (6.9%) did not have any treatment for HTN.

**Figure 1:**
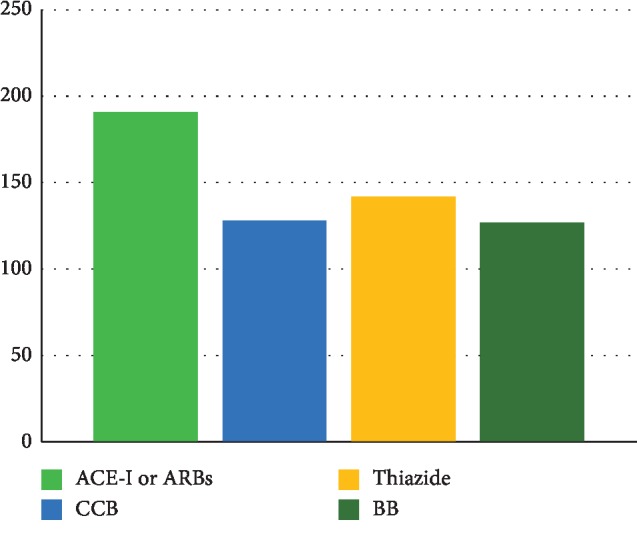
Distribution of the participant in the study according to the type of antihypertensive drug. ACE-I, angiotensin-converting enzyme inhibitor; CCB, calcium channel blocker; BB, beta blocker.

Figure 1 shows the distribution of participant according to the type of antihypertensive drug.

**Figure 2:**
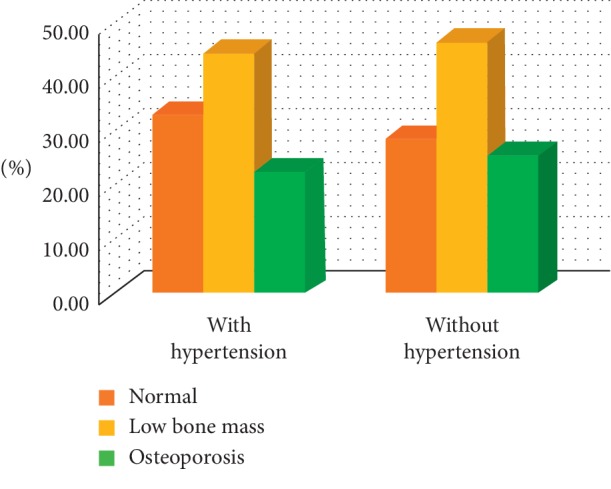
Distribution of low bone mass and osteoporosis between hypertensive and nonhypertensive participants.

The prevalence of osteoporosis and low bone mass were almost equal among women who had or did not have HTN, and no significant difference was found (*p*: 0.351) (Table 4 and Figure 2).

Independent-samples T-test was used to evaluate the relationship between the total hip BMD, the total lumbar BMD, and antihypertensive drugs.

The median total lumbar BMD was found to be increased significantly among women who took thiazide or beta blockers (BBs) compared with women who did not take either of them.  Table 5.

## 4. Discussion

HTN and osteoporosis are very common diseases among elderly population [6]. Many previous studies had suggested that HTN is an independent risk factor for fractures, but it was not clear if HTN affects BMD significantly [12].

The association between osteoporosis, HTN, and antihypertensive drugs was discussed in this study.

Among the 813 postmenopausal women, (45.2%) had low bone mass and nearly the fourth (24%) had osteoporosis. These results were in alignment with the results of many other studies in the Middle East and the west [14–20].

The prevalence of osteoporosis and low bone mass and osteoporosis increased significantly as the age and menopause duration prolonged, as previous other prospective studies showed that rates of bone loss from the spine and hip were 1% to 2% of its total mass during the early postmenopause phase and about 35% to 55% slower loss during the late menopause phase [21, 22].

A significantly negative relationship between BMI and the prevalence of osteoporosis was found in our study; this could be explained by the anabolic effect of adipokines secreted by adipose tissue [23, 24].

There was no association between HTN and osteoporosis according to the results of our study. This was consisted with the results of Fahad Javed F and colleague's study which found that the prevalence of low bone mass and osteoporosis were similar in those with or without HTN [25]. However, these results did not support the results of Li and colleague's meta-analysis, which demonstrated that HTN was associated with increased odds of having osteoporotic fractures, especially in women [26].

The exact mechanism underlying the effect of HTN on osteoporosis in humans has not been clear yet. Several mechanisms have been proposed. Although many studies had shown that high blood pressure is associated with increased loss of calcium in the urine, leading to a negative calcium balance of bone remodeling and increased levels of parathyroid hormone, which accelerate bone turnover and decrease bone mass [27, 28], recent studies had shown that hypertensive subjects had an increased levels of ghrelin [29, 30], which affect bone directly by inhibiting bone resorption and enhancing bone formation [31]. These counteracting mechanisms may ultimately lead to stabilization of BMD in hypertensive patients, which could explain the results of our study.

Moreover, antihypertensive drugs may have an add-on effect on bone. The results of our study showed that the use of thiazide or beta blocker drugs was significantly associated with increased levels of total lumbar BMD.

Several studies had investigated the effects of beta blockers on the bone to explore how beta blockers may improve BMD. Studies on animal models suggested that inactivation of the sympathetic nervous system impairs osteoclastic bone resorption and thus increases bone formation. [32]. And while many studies had not proven any improvements on BMD with beta blocker treatment [12, 33], others showed beneficial effects of beta blockers on osteoporotic subjects [33].

Similarly, thiazides may have an important role in preventing bone loss.

On one hand, thiazides decrease urinary calcium excretion by inhibiting the sodium chloride cotransporter in the distal tubule. [34]. And on the other hand, recent studies suggested that thiazides may have a direct effect on bone cells by enhancing osteoblast differentiation [35] and decreasing acid production through inhibition of carbonic anhydrase activity in the osteoclasts [34].

## 5. Conclusion

The results of our study show that there is no association between osteoporosis and hypertension in elderly postmenopausal Syrian women. However, our study shows a positive role of thiazide diuretics and beta blockers on the bone mineral density of the lumbar spine in osteoporotic hypertensive postmenopausal women. Further prospective studies with larger sample sizes and narrow age range are needed to evaluate the relationship between BMD and hypertension.

## Figures and Tables

**Table 1:** T-score values and diagnosis by the WHO.

Diagnosis	T-Score
Osteoporosis	*T* ≤ −2.5
Low bone mass	−2.5 < *T* ≤ −1
Normal	*T* > −1

**Table 2:** Description of numeric variables of the participants of the study.

Parameter	Mean ± standard deviation
Age	58.92 ± 8.6
Menopause duration	10.1 ± 8.3
Weight	77.09 ± 14.51
Height	152.16 ± 6.38
BMI	33.35 ± 6.13
Total lumbar BMD	0.905 ± 0.162
Total neck BMD	0.928 ± 0.148

**Table 3:** The general characteristics of the participants of the study (*N* = 813).

	Variable	Frequency	Percent (%)
Diagnosis	Normal	250	30.8
	Low bone mass	368	45.2
	Osteoporosis	195	24

Hypertension	Hypertensive	387	47.61
	Normotensive	426	52.39

BMI	Normal	59	7.3
	Overweight	188	23.1
	Obesity	566	69.6

Cigarette smoking	Yes	182	22.3
	No	631	77.7

Work	Yes	232	28.5
	No	581	71.5

Regular exercise	Yes	288	35.42
	No	525	64.57

BMI, body mass index.

**Table 4:** Distribution of low bone mass and osteoporosis among the participant according to their age, menopause duration, BMI, and hypertension diagnosis.

		Normal	Low bone mass	Osteoporosis	*p* value
Age group	40–50	52 (47.7%)	44 (40.4%)	13 (11.9%)	0.0001^†^
	50–60	129 (32.2%)	179 (44.6%)	93 (23.2%)	
	60–70	50 (23.3%)	108 (50.2%)	57 (26.5%)	
	>70	19 (21.6%)	37 (42%)	32 (36.4%)	

Menopause duration	<10	167 (38.7%)	186 (43%)	79 (18.3%)	0.0001^†^
	10–20	68 (22.5%)	152 (50.3%)	82 (27.2%)	
	>20	15 (19%)	30 (38%)	34 (43%)	

BMI	<25	1 (1.7%)	25 (42.4%)	33 (55.9%)	0.0001^†^
	25–30	46 (24.5%)	91 (48.4%)	51 (27.1%)	
	>30	203 (35.9%)	252 (27.1%)	111 (19.6%)	

Exercise	Yes	103 (35.8%)	126 (43.7%)	59 (20.5%)	0.046^†^
	No	147 (28%)	242 (46.1%)	136 (26.9%)	

Cigarette smoking	Yes	43 (23.6%)	80 (44%)	59 (32.4%)	0.004^†^
	No	207 (32.8%)	288 (45%)	136 (21.6%)	

Work	Yes	79 (34%)	103 (44.4%)	50 (21.6%)	0.367
	No	171 (29.4%)	265 (45.6%)	145 (25%)	

Hypertension	Yes	128 (33.1%)	172 (44.4%)	87 (22.5%)	0.351
	No	122 (28.6%)	196 (46%)	108 (25.4%)	

BMI, body mass index. †Statistically significant.

**Table 5:** Comparison of mean BMD among hypertensive patients according to the type of used antihypertensive drug.

		Mean of femoral neck BMD	*p* value	Mean of total lumbar BMD	*p* value
ACE-I or ARBs	No	0.926	0.726	0.899	0.08
	Yes	0.930		0.922	

CCB	No	0.930	0.146	0.904	0.907
	Yes	0.909		0.906	

Thiazide	No	0.926	0.653	0.899	0.019
	Yes	0.933		0.935	

BB	No	0.925	0.491	0.895	0.0001
	Yes	0.939		0.949	

BMD, bone mineral density; ACE-I, angiotensin-converting enzyme inhibitor; CCB, calcium channel blocker; BB, beta blocker.

## Data Availability

The data file (in EXCEL or SPSS) used to support the findings of this study are available from the corresponding author upon request.

## Abbreviations

BMD: Body mineral density;
BMI: Body mass index;
DXA: Dual-energy X-ray absorptiometry;
HTN: Hypertension;
ACE-I: Angiotensin-converting enzyme inhibitor;
CCB: Calcium channel blockers;
BB: Beta blockers.
